# Uses of Multi-Objective Flux Analysis for Optimization of Microbial Production of Secondary Metabolites

**DOI:** 10.3390/microorganisms11092149

**Published:** 2023-08-24

**Authors:** Marc Griesemer, Ali Navid

**Affiliations:** Lawrence Livermore National Laboratory, Biosciences & Biotechnology Division, Physical & Life Sciences Directorate, Livermore, CA 94550, USA

**Keywords:** secondary metabolism, flux balance analysis, COBRA, multi-objective flux optimization, systems biology, metabolic engineering, synthesis optimization

## Abstract

Secondary metabolites are not essential for the growth of microorganisms, but they play a critical role in how microbes interact with their surroundings. In addition to this important ecological role, secondary metabolites also have a variety of agricultural, medicinal, and industrial uses, and thus the examination of secondary metabolism of plants and microbes is a growing scientific field. While the chemical production of certain secondary metabolites is possible, industrial-scale microbial production is a green and economically attractive alternative. This is even more true, given the advances in bioengineering that allow us to alter the workings of microbes in order to increase their production of compounds of interest. This type of engineering requires detailed knowledge of the “chassis” organism’s metabolism. Since the resources and the catalytic capacity of enzymes in microbes is finite, it is important to examine the tradeoffs between various bioprocesses in an engineered system and alter its working in a manner that minimally perturbs the robustness of the system while allowing for the maximum production of a product of interest. The in silico multi-objective analysis of metabolism using genome-scale models is an ideal method for such examinations.

## 1. Introduction

Secondary metabolites (a.k.a. idiolites) are small, structurally diverse, chemical compounds that are generated by plants and microbes. They are produced by secondary metabolic processes during the stationary phase (idiophase) of an organism’s lifecycle that follows its growth phase (trophophase). Although these compounds are not essential for microbial growth, they confer a selective advantage to the organisms in their environments and serve many diverse and important functions. These include roles in bacterial survival and ecological interactions. For example, it has been shown that certain diatoms excrete secondary metabolites to promote the growth of beneficial bacteria and encourage direct physical interaction with them, while dissuading attachment by opportunist organisms and hindering their growth [1].

Secondary metabolites are also economically and medicinally important. Many herbicides, fungicides, bio-insecticides, antibiotics, immunosuppressants, antitumor drugs, and other high-value bioactive compounds are byproducts of microbial secondary metabolism. For example, certain alkaloid secondary metabolites (e.g., Taxol) have anticancer functions and can be produced by a number of different fungi, but the yield significantly varies between the different organisms [2].

Secondary metabolites are usually difficult to synthesize chemically. But, at times, this method is preferred over harvesting from biological sources. This is because chemical synthesis avoids problems like variable product quality that are associated with some natural systems [3]. However, the complexity of certain secondary metabolites (e.g., most alkaloid molecules) often makes chemical synthesis difficult or impossible. For such compounds, bioproduction and extraction is the most economically practical strategy [4]; to improve the harvest yield, there are many efforts underway to maximize the rate and efficiency of the metabolic processes that produce these compounds. While in many cases the organisms that are used for the production of secondary metabolites already have the metabolic machinery needed to produce these compounds (e.g., many members of *Actinomycetaceae*), sometimes—due to a desire for an increased growth rate or a need to couple the production of the compounds to the synthesis of other biochemicals—microbial chassis organisms such as *E. coli* are engineered to produce these compounds. The latter process could be riskier than using naturally producing organisms because at times the secondary metabolite or a byproduct of its production can end up being toxic to the chassis organism.

## 2. Optimization of Microbial Production of Secondary Metabolites

Often, the natural rates of production of secondary metabolites are too low to be industrially profitable. Thus, metabolic engineers spend a lot of time optimizing the rate of production of these compounds. One way of ensuring that a compound of interest is produced optimally is to accelerate the activity of primary metabolic pathways that produce the precursors and reducing the equivalents that are needed for its production [5]. Simultaneously eliminating competing processes that could siphon away energy, nutrients, or enzymatic capacity from the production of secondary metabolites also improves the yield and rate of production.

Our ability to manipulate complex biosystems is continually improving and the field of synthetic biology has made significant advances during the last decade. We are using heterologous gene expression to add functions to chassis organisms for the production of secondary metabolites (e.g., [6,7,8,9,10]). We also design novel synthetic gene circuits that encode new biological behaviors, dynamics, and control in our engineered systems (e.g., [11,12,13]). However, our control is still limited, and even when using microbial platforms whose workings we know best, we are at the mercy of stochastic fluctuations and other nonlinear interactions with our synthetically engineered circuitry that make it difficult to be fully sure of engineering outcomes.

While it might be possible to pinpoint a handful of obvious competing processes intuitively in model organisms, this will not be possible for system-level engineering. This is because most of our intuitive ideas stem from reductionist ideas and studies. For example, reductionist biochemical thinking would suggest that the overexpression of “rate-limiting” enzymes should result in significant increases in the activity of certain desired processes and this would lead to the increased production of one or more compounds of interest. However, this is not the observed behavior. To identify all of the processes that affect secondary metabolite production, and to predict changes that would optimally improve production rate and yield, requires system-level analyses using computational tools. System-level analyses have shown that the control of metabolic fluxes is not a single biocomponent property but instead a network property that is distributed across many enzymes [14,15,16] and is dependent on the topology of a network.

Manipulating complex biosystems to do our bidding is not trivial. This is because oftentimes we are forcing a system to operate in a manner that contradicts the regulatory rules that evolution has set forth for it. These regulations ensure maximum fitness for a system in its ecological niche. We might not be able to overcome these regulations. But if we have some understanding of a system’s most important objectives and how it prioritizes them under different environmental conditions, then we can devise ways to either work with the system’s regulations or “bend” them as much as possible to optimize our engineering objectives. The metabolic systems of microorganisms, even well-studied model organisms like *E. coli*, are too complex to learn about such tradeoffs using only reductionist methods and targeted analyses. Comprehensive in silico analyses are needed to fully grasp the multifaceted workings of complex biosystems.

## 3. System-Level Computational Models of Metabolism

**Figure 1:**
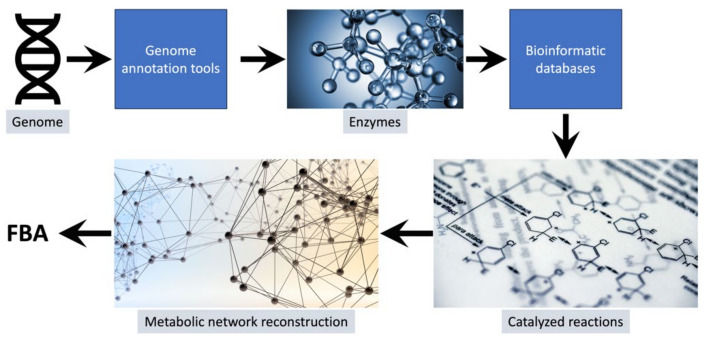
The process for developing genome-scale models of metabolism. The genome of the organism is annotated using genome-annotation tools such as RAST [33], Prokka [34], or KOALA [35]. The annotated genome provides a list of enzymes that can be used with bioinformatic databases such as KEGG [20,36], ModelSEED [21], and MetaCyc [22] to generate a list of all the reactions that can occur in the organism at different times. This list when further curated with empirical data and information from the literature provides a reconstruction of the metabolic network of the organism that can be used for FBA and other types of COBRA modeling.

System-level computational analyses are possible today due to revolutionary advances in high-throughput analytical technologies that permit the rapid and facile collection of multiple types of system-level data [17,18,19]. There are many different types of models that use process-appropriate mathematical formalisms to examine the operating principles of bioprocesses and systems in general. Some of the most widely used and successful methods for examining metabolic processes at a system-level are constraint-based reconstruction and analysis (COBRA) methods. These methods utilize omics data, particularly annotated genomes, to reconstruct the metabolic pathways that exist in a biosystem. The annotated genome provides a list of enzymes that an organism can produce. Using this list of enzymes with information found in biochemical databases such as KEGG [20], ModelSEED [21], and MetaCyc [22], a list of reactions that can be catalyzed within a system are compiled. This list is used to reconstruct the metabolic pathways in an organism and serves as the basis for genome-scale metabolic models (GEMs) (see Figure 1). Currently there are several tools available that allow for the rapid generation of draft GEMs for organisms [23,24,25,26,27,28]. The build metabolic model app available on the U.S. Department of Energy’s systems biology knowledgebase (KBase) is one such tool [29]. One helpful advantage of using KBase for developing draft GEMs is the availability of a unique app (merge metabolic annotations) that allows for the import and combination of genome annotations from multiple sources. This app can greatly help the process of curating draft GEMs [30,31] by ensuring that the tool returns a more complete network reconstruction with few orphan gap-filled reactions [32].

Detailed and accurate network reconstruction is particularly important for models that are used for studying organisms that produce secondary metabolites. This is because secondary and primary metabolism are tightly linked in these systems. The production of secondary metabolites requires precursor metabolites that are produced by primary metabolism. These include amino acids and short chain carboxylic acids. The precursors are used by proteins that are translated from groups of colocalized genes that work together to build the complex bioactive compounds. Polyketide synthases, non-ribosomal peptide synthases, and terpene cyclases are some of the most important classes of these biosynthetic gene clusters (BGCs). Most of the BGCs have characteristic catalytic domains that can be used to identify new gene clusters. This, along with the clustering characteristic of genes, has been used to develop computational tools (e.g., antiSMASH [37,38] and PRISM [39,40]) that can be used to identify BGCs from genome sequence data. These tools are critical for characterizing the secondary metabolism of organisms. The predicted biosynthetic capability can be used to build detailed GEMs that fully account for the connections between primary and secondary metabolism. The GEMs can then be used to identify the best candidate gene clusters for incorporation into engineered heterologous hosts.

### 3.1. Flux Balance Analysis

**Figure 2:**
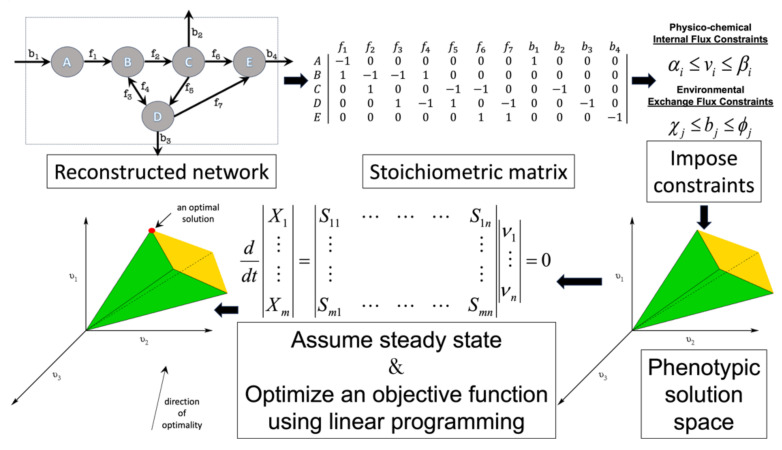
Flux balance analysis (FBA) is the most widely used COBRA analysis method. The genome-based metabolic network reconstruction is converted to the mathematically useful format of a matrix that details the stoichiometry of all the reactions in the system. The model is constrained using empirical data and fundamental physico-chemical laws. The system is assumed to operate at steady state and linear programming is used to solve for a feasible flux pattern that optimizes the activity of one biological objective.

Flux balance analysis (FBA) is a widely used mathematical method for using GEMs to study metabolism in biosystems. FBA finds optimal metabolic steady-state activity fluxes that satisfy constraints imposed by the metabolic network structure, mass balance, and the availability of nutrients (see Figure 2). FBA uses the annotated genome-based metabolic network reconstruction of a system. The reconstruction is gap-filled using empirical data and fundamental biochemical and biophysical laws. The metabolic reactions are mathematically represented by the stoichiometric matrix, *S* (*m* × *n*), where *m* is the number of metabolites and *n* is the number of different reactions. FBA operates on assumptions of mass balance and metabolic steady state. Based on these assumptions, the following set of linear equations govern the system’s behavior:

(1) $$\frac{dX_{i}}{dt}=\underset{j}{\sum }S_{ij}\nu_{j}=0$$

where *X_i_* is the concentration of metabolite *i*, *S_ij_* is the stoichiometry of metabolite *i* in reaction *j*, and *ν_j_* is the flux of reaction *j*. Other constraints that are imposed on the model based on experimental measurements limit the amount of nutrients that a system can import and byproducts that are exported. Also, if information regarding the average concentration of an enzyme and its catalytic turnover rate are available, flux through a reaction can also be constrained. Thus, in properly bound FBA simulations all reactions have a lower and upper boundary:

(2) $$\alpha \leq \nu_{j}\leq \beta$$

(3) $$\chi \leq b_{i}\leq \varphi$$

where *b_i_* is the export/import flux of species *i*; *α*, *β* are the lower and upper bound for internal fluxes; and *χ* and *φ* are the lower and upper limits for transport fluxes. For FBA simulations it is assumed that cells are at steady state. Balanced growth is assumed and a biomass reaction with fixed stoichiometric values is added to the model. This reaction quantifies the amounts of various metabolites that are needed to produce 1 g of biomass.

Once the GEM is appropriately constrained, FBA uses linear programming to solve for a feasible steady-state flux vector that optimizes an objective function. The most common objective function (cellular task to be optimized) is the production of biomass, i.e., cellular growth. However, other biological tasks can also be used as objective functions. There have been studies that have examined when and how such alternate objective functions might best be used [41,42]. Also, algorithms have been developed that use experimental data to infer the best objective functions for simulations [43,44]. Finally, as the use of FBA models expand into the realm of whole-cell modeling [45,46,47], variants of FBA have been introduced that loosen the rigid constraints imposed in classical FBA objective functions and bypass the limitations of steady-state and balanced growth assumptions [48].

FBA has been used to examine numerous biological topics that range from the fundamental nature of biological systems (e.g., [49,50,51]) to examining metabolism of deadly pathogens (e.g., [52,53,54,55]), studying cancer (e.g., [56,57,58,59]), elucidating the effects of genetic knockouts [60,61,62], and finding novel regulatory interactions [63]. The uses of FBA have continually increased. Automated tools for the generation of draft FBA models [21,23,24,25,26,27,28] have greatly helped this process. However, detailed analyses still require the use of human curated models. Even with advances in automated draft model generation, the overall process of developing high-fidelity models is still labor intensive [30,31].

Given the critical relationship between primary and secondary metabolism, system-level knowledge of the metabolic capabilities of an organism is critical for choosing the right biosystem for producing a secondary metabolite of interest. FBA studies are ideal tools for system-level analyses that can provide this information. In this vein, GEMs have been developed for several actinobacteria, particularly candidate members of the genus *Streptomyces* [64,65,66,67,68,69,70,71,72,73,74,75], a family of soil bacteria with diverse metabolisms that are known to produce a variety of different natural products, including anticancer drugs and antibiotics [76]. By using FBA with these GEMs, novel links between central metabolism and the production of secondary metabolites have been discovered. For example, it was found that the flux of nitrogen uptake and assimilation can positively affect the rate of antibiotic production in *Streptomyces coelicolor* [67,71]. Another study found that increasing pH can initiate the induction of idiophase in *Streptomyces peucetius* [66].

FBA has also been used to study the metabolic characteristics of the transition from active growth (trophophase) to idiophase. This transition is essential for the production of secondary metabolites because genes coding for the biosynthesis of secondary metabolites are usually not expressed at high growth rates [77]. The trophophase to idiophase transition is accompanied by an extensive rearrangement of cellular metabolism [72]. To gain a mechanistic understanding of these rearrangements, FBA was used to examine the flux patterns in the model secondary metabolite producer *S. coelicolor* during trophophase and idiophase [72]. FBA predicted that during the trophophase metabolites are mainly used for the production of biomass, while during idiophase the resources are shunted toward the production of secondary metabolites. The predicted flux pattern differences between the two phases were closely correlated with measured gene expression data. The correlation is so reliable that discrepancies between gene-expression data and predicted fluxes can be used to identify errors in genome annotation [72].

### 3.2. Multi-Objective Optimization

Optimizing the production and yield of a compound of interest usually requires engineering new strains of microbes. This is because natural selection has primed cellular metabolism for other biological objectives, such as growth and rapid adaptation to environmental pressures. The overproduction of compounds that are important to humans but have ancillary benefits for the biosystem are not prioritized. Cellular metabolism is tightly regulated to ensure against such “wasteful” overproduction of secondary metabolites. Therefore, efforts to overproduce secondary metabolites are often hindered by pathway competition with prioritized processes that are linked to the production of biomass components [78,79].

Given this divergence of biosystems’ and engineers’ objectives, engineering new idiolite-producing strains requires finding genetic manipulations that can work around the internal regulations of the systems while mitigating the fitness costs that may result from the induced perturbation. It is important to ensure that our engineered alterations do not drastically change the natural balance of biological objectives and subsequently the fitness of a system for a given environment.

GEMs can be powerful tools for identifying targets for such genetic manipulations. However, methods other than classical FBA need to be used. This is because FBA only optimizes one biological objective, while we need to examine the tradeoffs between multiple objectives. This is because evolution necessitates that organisms operate multiple critical processes at once and maximize the distribution of their limited resources in a Pareto-optimal fashion. A Pareto-optimal outcome is one where improvement in the performance of one task would result in diminishment of the ability to achieve (one or more) other tasks. To calculate Pareto-optimal solutions, examine the trade-offs between different objectives and identify bioprocesses that could hamper production or optimum yield of a desired product requires use of multi-objective (MO) optimization methods.

The multi-objective analysis of biological processes using constraint-based models is not a new method of analysis. One of the earliest such studies was the use of phenotype phase plane analysis (PPA) [80]. PPA was used to study the optimal uses of two model organisms’ metabolic networks as they adapt from variations of two environmental constraints [81,82]. Thus, PPA examined the tradeoffs between three system objectives (growth and the two constraints).

MO analyses are also preferred over plain FBA for optimizing the production of desired byproducts. Again, this is because FBA only optimizes one objective function, and unfortunately the usually optimized growth objective is often not appropriate for studying the production of natural products. For example, an analysis of the production of certain bioactive compounds by *Streptomyces clavuligerus* showed that the maximum ATP yield is the best objective function and that neither the maximization of growth nor bioactive compounds would agree with the experimental data [75]. If the objective that is optimized by FBA is growth, then the analysis will primarily examine the primary metabolism. It will ignore the secondary metabolism and production of compounds that are not biomass components. To overcome this shortcoming of FBA, several MO optimization methods have been developed to optimize the production of compounds of interest. We discuss some of them below.

#### 3.2.1. OptKnock

**Figure 3:**
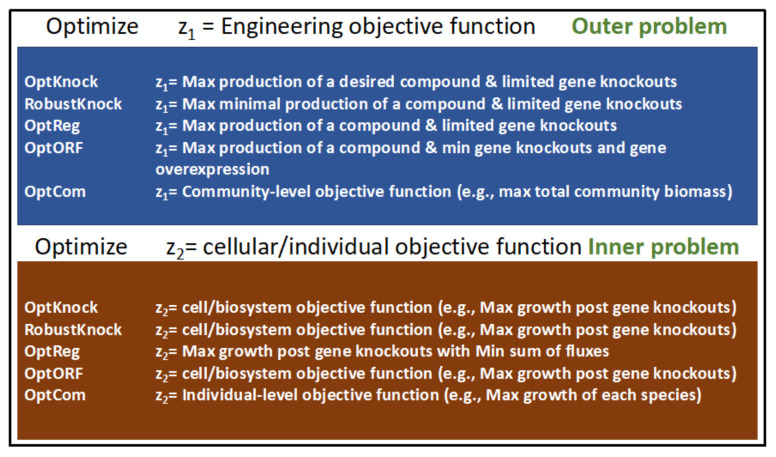
A list of some bilevel optimization programs that have been developed for designing microbial strains that maximize the production of compounds of interest. The outer and inner optimization problems for each tool are listed.

One of the earliest MO optimization methods developed for strain engineering is OptKnock [83]. OptKnock is a bilevel optimization tool that uses GEMs and mixed-integer linear programing (MILP) to suggest gene manipulation strategies that result in the overproduction of a compound of interest. OptKnock achieves this by aligning the engineering objectives with the internal objectives of a biosystem. Thus, the proposed alterations work with the system instead of against it. OptKnock accounts for the fact that the metabolic flux distribution is controlled by system-specific internal objectives and that the surest way of ensuring a process is active is to make it essential for optimizing primary cellular objectives. To this end, OptKnock proposes genetic manipulations that make the production of the desired compound essential for cellular growth. For typical OptKnock simulations the maximization of biomass production (greater than a preset minimal level) is treated as the primal optimization while a dual optimization problem solves for reaction knockouts that maximize production of the compound of interest within the constraints set forth by optimization of primal problem (See Figure 3).

OptKnock has been used for many strain design studies where the maximum production of a compound of interest was the goal (e.g., [84,85]). OptKnock has subsequently been updated with several variants. RobustKnock [86] expands upon OptKnock by identifying and eliminating competing pathways that could divert flux away from the production of compound of interest. OptReg [87] is a bilevel optimization platform that predicts the gene expression/enzyme level adjustments that could lead to the increased production of a compound of interest. OptORF [88] is another bilevel optimization platform that predicts engineering strategies using combinations of gene knockouts, differential gene expression, and the manipulation of regulatory pathways. OptFlux [89] is an open-source computational systems biology software that identifies engineering targets using evolutionary algorithms and simulated annealing. These meta-heuristic methods can work with different types of objective functions, including nonlinear ones. OptFlux also allows for the use of OptKnock for strain optimization.

#### 3.2.2. OptStrain

OptStrain [90] is a major upgrade to OptKnock for designing new strains. This is because, unlike the other version of OptKnock, OptStrain can identify non-native genes/enzymes that can be incorporated into a system in order to increase the production of a desired compound. To inform OptStrain of the universal reactome, the developers of OptStrain compiled a regularly updated large database of biochemical reactions. OptStrain initially identifies a maximum yield pathway for the production of the desired compound from a selected substrate. This step is not species specific and uses all reactions deposited in the reaction database. OptStrain then uses combinatorial optimizations to search for stoichiometrically balanced pathways that include the minimum number of non-native reactions while satisfying the aim of maximizing the yield of the product. If GEMs are available for multiple host organisms, OptStrain can also be used to choose the best host for engineering projects. Once the set(s) of reactions/genes that need to be added to a system are identified, OptKnock can be used to predict the gene knockouts that will couple production of the compound of interest to cellular growth.

Bilevel optimization methods have been some of the most popular tools for strain engineering during the past two decades and are still being used for designing microbial strains that optimize the production of valuable products (e.g., [91,92]). Figure 3 lists some of the prominent bilevel optimization tools and their inner and outer objective functions.

#### 3.2.3. MultiMetEval

**Figure 4:**
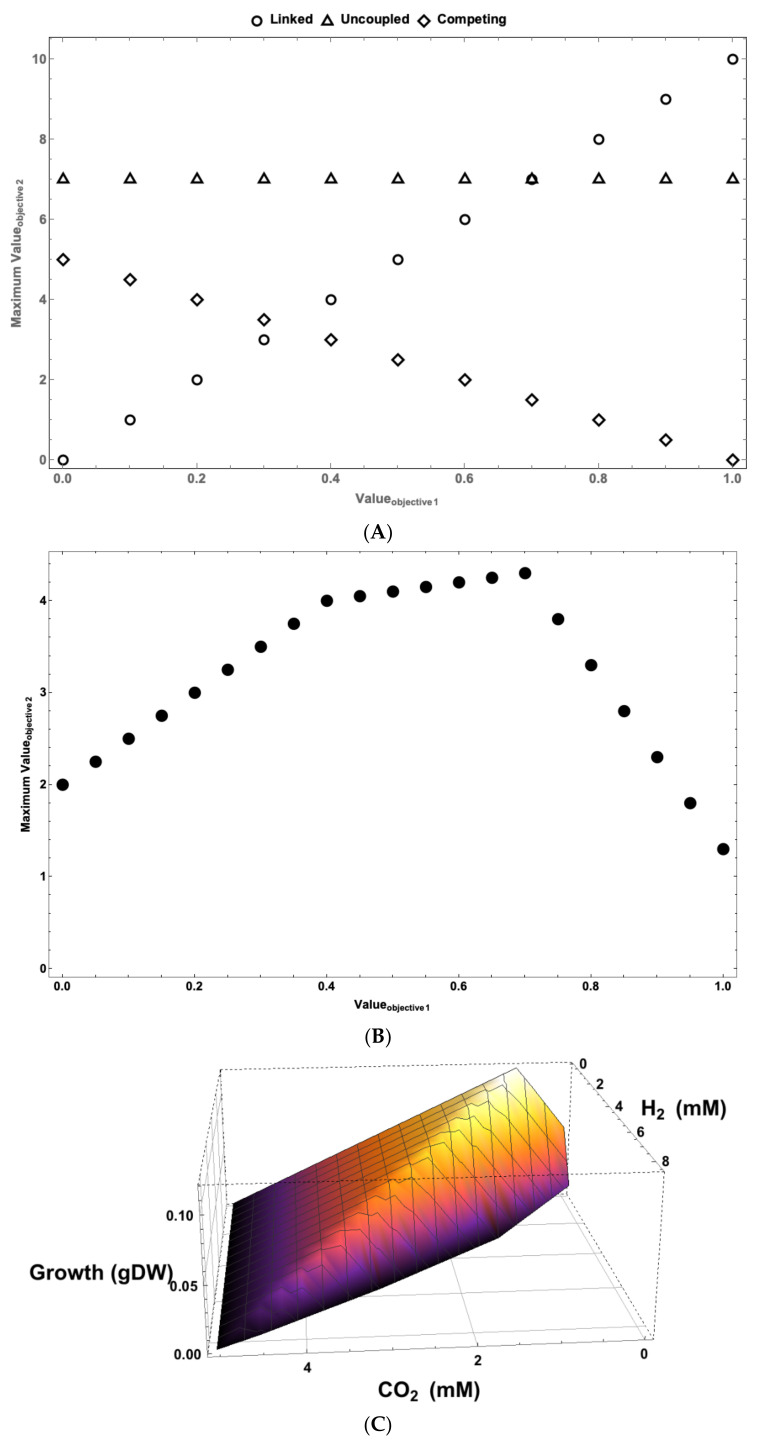

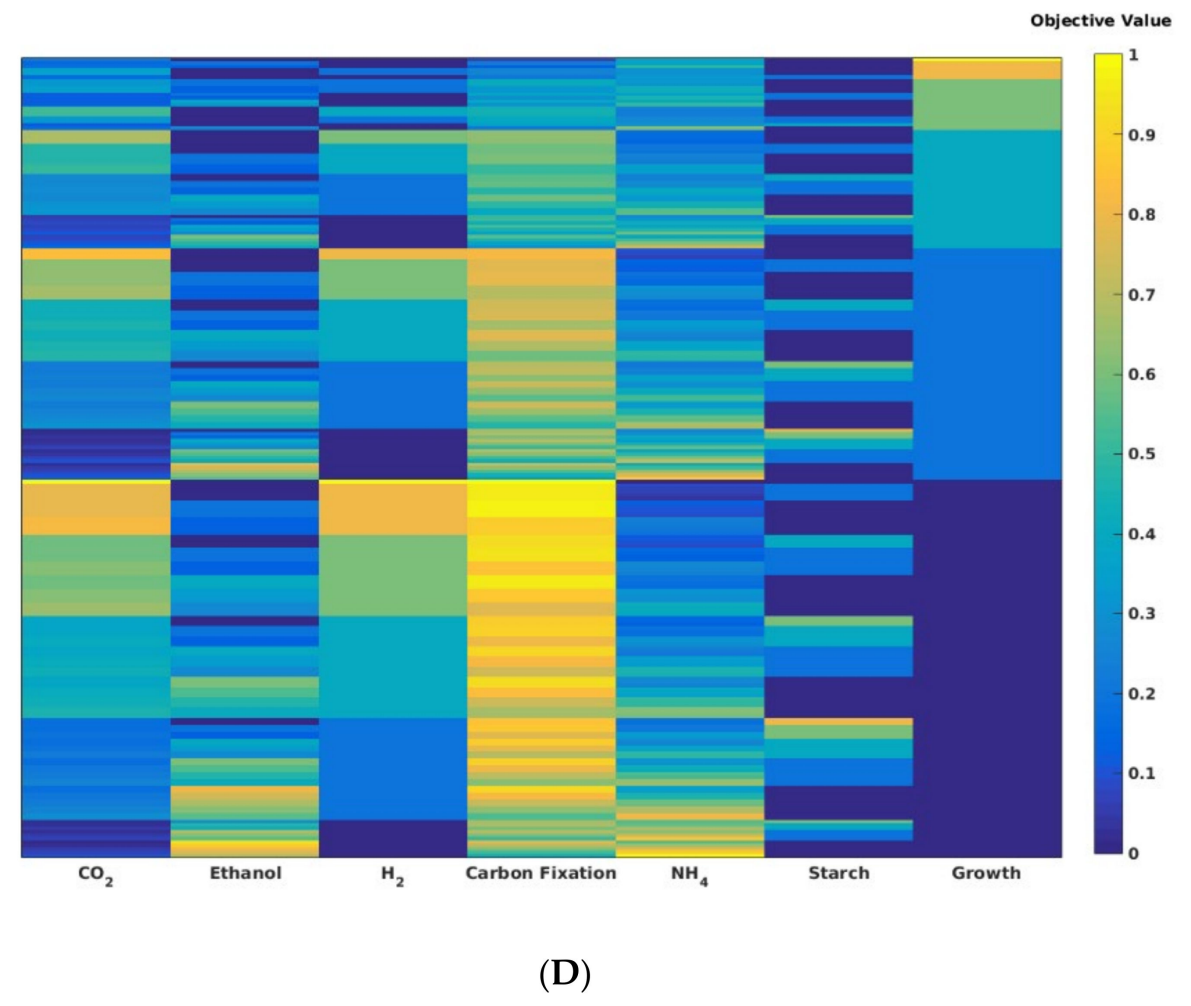
Pareto fronts provide a wealth of information about the nature of interactions between different system objectives. (**A**) The 2D Pareto fronts show the nature of interactions between two system objectives. The interactions between objectives can be linked (improvement in one requires improvement in the other), uncoupled (value of one has no effect on the value of the other), or competing (increase in value of one lowers the value of the other). (**B**) In complex systems, the nature of interactions between two objectives can change depending on their values. Such multiphasic interactions can greatly help systems adapt to changes. Pareto fronts (depending on the number objectives that have been examined) can be visualized in a variety of different ways. (**C**) The 3D representation of a Pareto front can be used for visualizing the outcome of analysis from methods like PPA. This figure shows tradeoffs between hydrogen production, carbon fixation, and growth in *Rhodopseudomonas palustris* (based on results from Navid et al. [94]). (**D**) For analyses beyond three dimensions, heatmaps can be used to visualize the results. Here, a heatmap representing the Pareto front resulting from a seven-dimensional MOFA analysis of metabolism and biofuel production in *Chlamydamonas reinhardtii* is shown.

MultiMetEval [93] is an MO optimization tool that utilizes COBRA methods to calculate the Pareto front between two cellular objectives. A Pareto front (see Figure 4) is a collection of Pareto-optimal solutions. The process of calculating a two-dimensional Pareto front (see Figure 4A,B) involves calculating the maximum ($\varpi_{1}^{max}$) and minimum value of the first objective ($\varpi_{1}^{min}$) using a GEM and FBA. The GEM is then updated with $\varpi_{1}^{max}$ as a fixed value for the first objective. The updated model is then solved for the optimum value of the second objective (ϖ_2,0_) when the first objective operates at $\varpi_{1}^{max}$. This is one Pareto-optimal solution and one point on the Pareto front. If one wants to map the surface of the Pareto front using P points (including the one calculated at $\varpi_{1}^{max}$), the GEM is constrained P − 1 times in an iterative fashion by the value:

(4) $$\varpi_{1,p}=\varpi_{1}^{max}-\frac{p\times \Delta }{P-1}$$

(5) $$p=\left\{1,\cdots ,P-1\right\}$$

(6) $$\Delta =\varpi_{1}^{max}-\varpi_{1}^{min}$$

For each ϖ_1,*p*_ value, the optimum value of the second objective (ϖ_2,*p*_) is calculated.

Two-dimensional Pareto fronts provide a wealth of information about the nature of interactions between two processes (objectives) in a system. As can be seen from Figure 4A, the interactions between objectives can be uncoupled, i.e., changes in the value of one would not affect the value of the other. They can be fully linked, i.e., any improvement in the operation of one requires an improvement in the value of the other. Alternatively, they could be competing, where any increase in the activity of one objective leads to a reduction in activity of the other. In most complex systems, the tradeoffs between objectives are not as simple as what is shown in Figure 4A. Instead, in most complex systems, the tradeoffs are multiphasic (see Figure 4B). This means that at different values for Objective 1, the nature of its interaction with Objective 2 changes. This could mean that the nature of interaction of an objective with another objective could change depending on its activity. For example, it could switch from being positively coupled to the other objective to competing with it (see the difference between Phases 2 and 3 in Figure 4B).

MultiMetEval also provides a platform to comparatively analyze metabolic differences between multiple GEMs. This can be used to identify organisms that are naturally adapted to overproducing secondary metabolites. The tool was used to examine the production capability of a large group of actinomycetes for different classes of secondary metabolite [93]. The study resulted in a number of interesting finds. One of the most intriguing finds was that organisms that have the most productive and metabolically versatile metabolisms are not the ones that are being used for biotechnology. Additionally, it was found that genome size and the complexity of secondary metabolism do not correlate with an organism’s productivity.

#### 3.2.4. Multi-Objective Flux Analysis (MOFA)

While FBA studies typically optimize growth as the cell’s primary objective [42], it has been shown that no single objective fully governs the behavior of a system. A study examining the tradeoffs between double and triple combinations of various objectives in microbes showed that a Pareto-optimal combination of three primary tasks—maximum biomass yield, maximum yield of ATP, and the optimal allocation of resources—best describes the measured flux distribution for a variety of organisms and conditions [51]. However, the fluxes predicted for Pareto-optimal combinations of the primary objectives do not exactly match with experimental results. The Pareto optimization of other biological objectives that might be important for specific organisms and/or growth conditions could help to reduce the observed mismatch. The examination of these “secondary” objectives could also provide a quantitative measure of how these processes control the operation of a cell. This knowledge will help guide synthetic biology and metabolic engineering efforts so that the rates and yields of secondary metabolites are increased.

MO methods have been developed to study metabolic tradeoffs in systems that produce a variety of different high-value commodities such as biofuels (e.g., [95,96,97,98,99]). We recently developed a curated GEM for *Rhodopseudomonas palustris* [94], a metabolically versatile purple non-sulfur bacterium that has been studied as a model organism for the production of hydrogen gas (H_2_) [100,101,102]. To gain a better understanding of the tradeoffs between the different biological objectives (e.g., growth, ATP production, H_2_ production, carbon fixation, metabolism of aromatic compounds), we developed an MO analysis tool that we call multi-objective flux analysis (MOFA). Similar to Nagrath et al. [103], MOFA uses the normalized normal constraint (NNC) [104] method for MO analyses. This ensures that MOFA’s output is an *n*-dimensional (*n* = number of objectives) Pareto front comprised of a set of evenly distributed Pareto-optimal points, regardless of differences in the magnitudes of the examined objectives.

We developed MOFA because we wanted to examine the tradeoffs between more than a handful of objectives. Our high-dimensional (7 and 8 objectives) analyses provided us with novel insights into *R. palustris’* ability to produce H_2_ as a biofuel. As with the production of secondary metabolites, we found that the production of H_2_ under all conditions drastically reduces the organism’s growth rate [94].

We have since developed a Matlab version of MOFA that can be used with the COBRA toolbox [105]. The COBRA toolbox is one of the most widely used platforms for computational systems-level analyses. We think this code would be useful for users of the COBRA toolbox who are interested in conducting high-dimensional MO analyses. Thus, we are including this code as a supplementary addition to this manuscript. A user guide is also included with the Supplementary Materials.

### 3.3. System-Level Analysis of Microbial Communities

Given that secondary metabolites have a major role in how microbes interact with their surroundings, engineering microbial communities can be one way of inducing the production of secondary metabolites [106,107,108,109]. Changing the dynamics of multicellular and multispecies systems by various means such as: altering the interactions between constituents via engineered loss or gain of functions, adding or reducing a member’s metabolic burden, or simply changing the community’s growth environment can result in changes in the secondary metabolism of individual organisms and the community as a whole. For example, one study has shown that dividing the metabolic pathways for the production of the chemotherapeutic compound paclitaxel improved its production [110]. As another example, it has been shown that in microbial cocultures presence of organisms that lower concentrations of metabolites that inhibit growth of another community member can greatly boost overall community growth rate [111]. In case of production of secondary metabolites, it has been shown that glucose interferes with biosynthesis of secondary metabolites [77]. So, addition of an organism that is a voracious consumer of glucose and producer of alternate forms of carbon could be a possible community engineering strategy for improving production of secondary metabolites.

#### 3.3.1. OptCom

One MO-based tool developed for examining interactions in microbial communities is OptCom [112]. OptCom was developed by the same group that also developed OptKnock [83] and OptStrain [90]. As with these programs, OptCom utilizes bilevel optimization to examine the tradeoffs between individual vs. community fitness objectives in a multispecies microbial community. This allows for a quantitative and directed examination of the metabolic roles of each species in a community and the overall ecological niche. The nested bilevel optimization problem in OptCom is formulated using species-level objectives as the inner optimization while the community-level objective is optimized as the outer optimization (see Figure 3). Each species in the modeled community has its own biomass equation which is separately optimized in the inner level. The interactions between the constituent members of the community are constrained by limits on the exchange of metabolites between species. Community-level operation, such as the production of total community biomass, is optimized as the outer problem.

OptCom has been used to examine different types of interactions (positive, negative, or neutral) in microbial communities. This can be achieved by adjusting the inter-species flux constraints, i.e., varying the community-level optimization problem. A subsequent update to OptCom named d-OptCom [113] allowed for the simulation of transient changes in biomass of each community member. d-OptCom also allows changes to the concentrations of metabolites in the shared growth medium.

#### 3.3.2. Community and Systems-Level Interactive Optimization (CASINO)

CASINO [114] is another bilevel optimization program that has been developed for the examination of interactions in complex multicellular and multispecies communities. CASINO uses community network properties to define the topology of a community. The inner problem is the optimization of biomass for individual species, while the outer problem is the optimization of biomass with the added aim of the optimum distribution of resources between species. The method was used to extensively study the interactions between the human gut microbiome and the host under different dietary regimens.

## 4. Conclusions

Genome-scale models of metabolism and COBRA methods have become indispensable tools for system-level analyses and metabolic engineering. COBRA methods have been used widely to study the metabolism of microbes. They have provided novel insights that have been used to engineer new microbial strains that have high production rates for commercially, industrially, and medicinally important bioactive compounds. For example, FBA simulation methods have been used with high-performance computing and pathway-search algorithms to predict putative heterologous biosynthesis pathways for over 6000 compounds in 70 different microbes [115].

While bilevel optimization tools and MO-based methods have been some of the most often-used tools for synthetic biology and metabolic engineering, other types of constraint-based modeling informed with different omics data could also contribute to these efforts. For example, it has been shown that metabolic flux changes associated with the transition of cellular metabolism from growth to idiophase closely correlate with gene expression dynamics. This provides a possible route for examining secondary metabolism in microbes by constraining GEMs with gene-expression data. Many tools have been developed for this purpose (e.g., [54,116,117,118,119,120,121,122]), and their uses and differences have been reviewed [123,124,125]. The deluge of heterogenous system-level data makes the use of these types of modeling essential for elucidating the metabolic state of a system under different conditions, particularly when the change in the environment is not biochemical but physical (e.g., temperature change). Beyond GEMs, the development and use of detailed whole-cell models [46,47] that account for the activity of every molecule in a system can greatly expand the role of computational models in the analysis of organisms capable of producing high-value bioactive compounds.

## Supplementary Materials

The following supporting information can be downloaded at: https://www.mdpi.com/article/10.3390/microorganisms11092149/s1. Code and manual for Matlab version of MOFA. References [104,105,126,127,128,129] are cited in the Supplementary Materials.
