# Supplementary material for: Uses of Multi-Objective Flux Analysis for Optimization of Microbial Production of Secondary Metabolites
Source: Microorganisms. 2023 Aug 24;11(9):2149. doi: 10.3390/microorganisms11092149 (PMC10536367; doi:10.3390/microorganisms11092149)
Supplement: Supplementary file 1 [file microorganisms-11-02149-s001.zip › MOFA-User-Manual.pdf]

MOFA User Manual

Marc Griesemer Ali Navid

Table of Contents

1. Introduction to MOFA 2

2. User Requirements 2

2.1Hardware and software requirements 2

2.2 Simulation requirements 2

2.2.1 User Considerations 2

2.2.2 Structure of Input Arguments 2

2.2.3 Runtime Factors 2

3. Installation 3

4. MOFA Function Structure 3

4.1 Index of MOFA functions 3

4.2 Functions for running a simulation 3

4.2.1 mofa.m 3

4.2.2 min_max.m 3

5. An example MOFA analysis using an E. coli model 4

6 Normalized Normal Constraint (NNC) method 5

7. Definitions 8

References 8

1. Introduction to MOFA

MOFA is a COBRA toolbox (1-3) extension using MATLAB® for performing multi-objective analysis of trade-offs among different objectives of a biosystem using constraint-based models of biochemical processes. This document outlines the software. Any queries regarding use of MOFA are welcome and should be directed to Ali Navid (navid1@llnl.gov).

2. User Requirements

2.1 Hardware and software requirements

No specific hardware requirements are necessary beyond what MATLAB needs. The code uses the GLPK solver that is included with the latest download of COBRA Toolbox. No other LP solvers are currently supported.

2.2 Simulation requirements

2.2.1 User Considerations

1. The user needs to ensure that model is well constrained and provides accurate FBA solutions that do not violate mass balance and thermodynamic laws.
2. The user must confirm that the model would be valid (without errors) for optimization using COBRA toolbox.

2.2.2 Structure of Input Arguments

MOFA needs information on the parameters of the simulation: namely, the model, the number of divisions of the Pareto front, and the specified objectives. The user can use an input file (see Table M1) or function arguments to supply the list of objectives. The program uses a modified COBRA model object that has unique constraints for the defined objectives. The solution is the *n*-dimensional (*n*=number of examined objectives) solution of the normalized objective values that composes the Pareto front. One must use valid objectives contained in the model; otherwise, the program will print an error message and exit. It is useful to check that the objective names are correctly spelled and that there are no blank lines at the end of the list.

2.2.3 Runtime Factors

The time taken by the simulation depends on:

1. The number of objectives
2. The number of divisions
3. The objectives chosen.

Table M1: Structure of input file includes list of objectives, including the main objective

(name of the file).txt
COMMENT<br>(name of the objective to be optimized during MOFA iterations)<br>#COMMENT<br>(objective name 1)<br>(objective name 2)<br>...<br>(objective name n)

3. Installation

1. Install MATLAB® (http://www.mathworks.com); a license is required.
2. Download and install the COBRA toolbox as directed from https://opencobra.github.io/cobratoolbox
3. Install MOFA by adding the package folder into the COBRA folder. Alternative locations outside the MATLAB path for MOFA will require a path addition.

4. MOFA Function Structure

4.1 Index of MOFA functions

1	mofa.m	Main function
2	min_max.m	Finds the minimum and maximum fluxes of all objectives
3	anch_pts.m	Finds the anchor points, auxiliary function

4.2 Functions for running a simulation.

There are the two functions the user can use to conduct MOFA analyses, mofa and min_max. A third function, anch_pts, determines the anchor points but is an auxiliary function used only by the main MOFA program. This subsection gives a listing of the inputs and outputs of these functions.

4.2.1 mofa.m

This is the main MOFA function for the code.
function [mofa_sol, mxhr, mihr, aphr] = ***mofa***(model, inp_file, [], [], ndiv, mi_mx)

Inputs:

model	COBRA model object
inp_file	Input text filename with listing of objectives (string)
ndiv	Optional: number of divisions (integer)
mi_mx	Optional: Main objective minimized or maximized (string, 'min' or 'max')

Outputs:

mofa_sol	List of feasible values for Pareto points (matrix of doubles)
mxhr	*n* member array (doubles) containing the calculated maximum values of each objective.
mihr	*n* member array (doubles) containing the calculated minimum values of each objective.
aphr	2-D matrix (doubles) containing the anchor points. Its size is ($n \times n$).

4.2.2 min_max.m

This function is used by the main MOFA function but can be called independently.

function [mxhr,mihr] = ***min_max***(model, objc)

This function solves for the maximum and minimum fluxes of each objective. This is another way of conducting flux variability analysis (FVA) (4). FVA is a method in COBRA that solves for

the upper and lower bounds of all steady-state reaction fluxes in a model. In the MOFA code, this is done only for the objectives of interest.

Inputs:

model	COBRA model object
objc	the names of the objectives (cell array of strings)

Outputs:

mxhr	*n* member array (doubles) containing the calculated maximum values of each objective.
mihr	*n* member array (doubles) containing the calculated minimum values of each objective.

5. An example MOFA analysis using an *E. coli* model

First, initiate the COBRA toolbox:

>> initCobraToolbox

GLPK is the only supported solver at this time. If it is not the current solver, then the program will switch to it for MOFA usage.

The E. coli model (iAF1260) (5) can be downloaded from:

https://www.embopress.org/action/downloadSupplement?doi=10.1038%2Fmsb4100155&file=msb4100155-sup-0006.zip

unzip the file and from the folder *msb4100155-sup-0006* copy the file *Ec_iAF1260_flux1.txt* to your COBRA folder. Change the designation of the file from .txt to .xml

We chose iAF1260 as an example because it is a widely used human-curated model. The example MOFA analyses are meant solely to demonstrate the workings of our MOFA code, rather than to provide novel biological insight.

The model should be imported to a COBRA model object.

The model can be loaded into MATLAB using the following command:

>> model = readCbModel('Ec_iAF1260_flux1.xml');

Then navigate to the directory where the MOFA folder is located.

The inputs for the MOFA analysis should be placed in an input file. The file "*mofa_Ecoli_sample_input.txt*" has been included with our code.

For this analysis we examine 6 different objectives. The growth main objective (obf = Ec_biomass_iAF1260_core_59p81M) and five other objectives (obj = {EX_o2_e_, EX_co2_e_, EX_ac_e_, EX_etoh_e_, EX_nh4_e_} representing exchange reactions for oxygen, carbon dioxide, acetate, ethanol, and ammonia, respectively.

The input file for the *E. coli* simulation with 6 objectives is shown in Table M2.

For a full list of available reactions, examine the 'rxns' and 'rxnNames' fields in the COBRA model object.

Finally, the number of divisions can also be specified:

>> ndiv = 5;

The number of divisions must be positive and greater than 3.

Table M2: Input file for the *E. coli* with objectives chosen from the iAF1260's reactions ('*mofa_Ecoli_sample_input.txt'*).

```
#enter the name of the objective to be optimized
Ec_biomass_iAF1260_core_59p81M
#enter the list of other objectives
EX_o2_e_
EX_co2_e_
EX_ac_e_
EX_etoh_e_
EX_nh4_e_
```

At this point, one can look at the model object and make modifications to the constraints and other fields as necessary before calling the main MOFA function.

The MOFA function command is:

```
>> [mofa_sol, mxhr, mihr, aphr] = mofa(model,'mofa_ecoli_input.txt'
, [] , [] , ndiv, 'max');
```

To review, the first argument is the model as a COBRA object; the second is the name of an input file containing the list of objectives; the third is the list of objectives; the fourth gives the main objective but since both are included in the input file, they are blank. The fifth and sixth are the number of divisions and whether to maximize ('max') or minimize ('min') the main objective, respectively. Only the first two arguments are mandatory. The last four are optional. If the number of divisions and optimization sense are not supplied, the default number of divisions is 10 and the main objective will be maximized.
A file showing the output of an example MOFA simulation (ndiv=5, input file= mofi_ecoli_input.txt) titled "mofa_output.txt" is included. It lists the 1512 Pareto optimal solutions that form the 6D Pareto front at a relatively low level of granularity. The results are normalized, i.e., the fraction of the optimum value that the objective can attain given the model constraints. Thus, the values will be between 0 and 1. All of the output values are provided both in a tab-separated data file and in MATLAB variables for further analysis and graph plotting using MATLAB or Excel.

6 Normalized Normal Constraint (NNC) method

Our MOFA code uses the NNC method of multi-objective optimization (6).
Problem Statement: The multi-objective optimization (MO) problem can be defined as:

$$\min_{x}\{F_1(x)F_2(x)\cdots F_n(x)\}, n \geq 2 \qquad (1)$$

Subject to the constraints:

$$g_j(x) \leq 0, 1 \leq j \leq r \qquad (2)$$

$$h_k(x) \leq 0, 1 \leq k \leq s \qquad (3)$$

$$x_{li} \leq x_i \leq x_{ui}, 1 \leq i \leq n_x \qquad (4)$$

The vector *x* denotes the set of constraint variables and F_i denotes the *i*th objective.

Algorithm Steps:

Step 1: Initialize Simulation

a. Load SBML model or COBRA model object.
b. Process input file or lists of objectives.

Step 2: Find utopia/nadir points (maximum/minimum flux values for all objectives).

The points that contain the set of maximum and minimum values of all objectives are called the

1. Utopia point
$$F^U = [F_1(x^{1*})F_1(x^{2*}) \cdots F_1(x^{n*})] \qquad (5)$$
2. Nadir point
$$F^N = [F_1^N F_2^N \cdots F_n^N] \qquad (6)$$
Where $F_i^N = max[F_1(x^{1*})F_1(x^{2*}) \cdots F_1(x^{n*})], i \in \{1,2,\cdots n\}$.

These points are used as reference points in objective space as there is not a way to simultaneously optimize all objectives. In the NNC, the utopia point is used to normalize the space in each dimension. This is essentially performing Flux Variability Analysis (FVA) on the defined objectives. The difference between these two points gives the range for each dimension in objective space, a vector:

$$\bar{F} = \begin{Bmatrix} \nu_1 \\ \vdots \\ \nu_n \end{Bmatrix} = F^N - F^U \qquad (7)$$

Which leads to the normalized objectives,

$$\bar{F_i} = \frac{F_i - F_i(x^{i*})}{\nu_i}, i \in \{1,2,\cdots n\} \qquad (8)$$

Step 3: Find Anchor Points

The NNC method uses anchor points as reference vertices in objective space. The anchor points are calculated by individually minimizing each objective (F_j) individually, subject to the problem constraints, to obtain the jth anchor point, F_j ($j = 1,\ldots,n$).

Step 4: Define utopia line (utopia hyperplane)

From the vertices of the anchor points, we can define a utopia hyperplane.
We define the direction of the utopia line vector

$$\bar{N} = \bar{F}^{n*} - \bar{F}^{k*} \qquad (9)$$

Then we compute a normalized increment along the direction $\bar{N}_k$ for a prescribed number of divisions *x* for each direction *k*:

$$\delta_k = \frac{1}{x-1}, 1 \leq k \leq n-1 \qquad (10)$$

Parameter α_{ij} is incremented by δ between 0 and 1 and we use values of j where $j \in \{1,2,\cdots,n\}$.

Step 5: Generate evenly distributed hyperplane points.

Evaluate a set of evenly distributed points on the Utopia hyperplane as

$$X_{pj} = \sum_{j=1}^{n} \alpha_{jk} \bar{F}^{k*} \qquad (11)$$

where

$$0 \le \alpha_{jk} \le 1 \qquad (12)$$

and

$$\sum_{k=1}^{n} \alpha_{jk} = 1. \qquad (13)$$

Next the use the set of equally distributed points generated at the previous step to compute the Pareto solution by solving the following LP problem at each point individually

$$min\, \bar{F}_n \qquad (14)$$

$$g_j(x) \le 0, 1 \le j \le r \qquad (15)$$

$$h_k(x) \le 0, 1 \le k \le s \qquad (16)$$

$$x_{li} \le x_i \le x_{ui}, 1 \le i \le n_x \qquad (17)$$

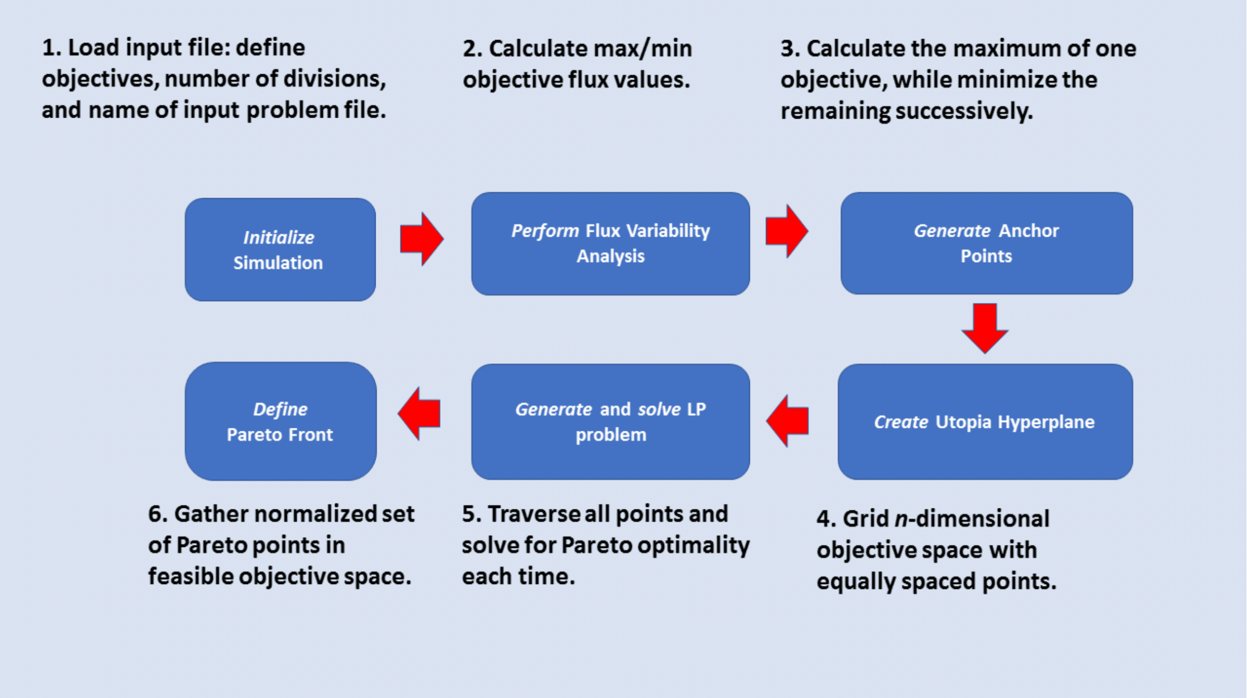

Figure M1: Schematic of MOFA workflow

Step 6: Gather Pareto points to form Pareto frontier.

The set of Pareto points is combined into the frontier.

Step 5 is performed repeatedly, traversing from point to point until all are covered. The simulation also skips points close to the previous point. Figure M1 summarizes and categorizes the workflow of the current version of the software.

7. Definitions

Flux variability analysis (FVA): for a given level of the cellular objective (e.g., biomass yield) finding the upper and lower bounds of all steady-state reaction fluxes can be determined.

Anchor point: Axis point in multi-objective space where the objective interest is at its maximum.

Utopia (hyper)plane: the multidimensional plane formed by the connection of the anchor points.

Pareto front(ier): The set of feasible points in objective space at points where moving away from it improves the value of the others.

COBRA Toolbox: Constraint-Based Reconstruction and Analysis add-on to MATLAB®.

Normalized Normal Constraint (NNC) method: a multi-objective algorithm for generating an equally spaced set of Pareto points.

*Feasible: Feasible solution found.

*Wasted Simulation: Infeasible or unbounded solution found at point.

* Definition refers to variables in the code.

References

1. Heirendt L, Arreckx S, Pfau T, Mendoza SN, Richelle A, Heinken A, et al. Creation and analysis of biochemical constraint-based models using the COBRA Toolbox v. 3.0. Nature protocols. 2019:1.
2. Schellenberger J, Que R, Fleming RMT, Thiele I, Orth JD, Feist AM, et al. Quantitative prediction of cellular metabolism with constraint-based models: the COBRA Toolbox v2. 0. Nature protocols. 2011;6(9):1290-307.
3. Becker SA, Feist AM, Mo ML, Hannum G, Palsson BO, Herrgard MJ. Quantitative prediction of cellular metabolism with constraint-based models: the COBRA Toolbox. Nat Protoc. 2007;2(3):727-38.
4. Mahadevan R, Schilling CH. The effects of alternate optimal solutions in constraint-based genome-scale metabolic models. Metab Eng. 2003;5(4):264-76.
5. Feist AM, Henry CS, Reed JL, Krummenacker M, Joyce AR, Karp PD, et al. A genome-scale metabolic reconstruction for Escherichia coli K-12 MG1655 that accounts for 1260 ORFs and thermodynamic information. Mol Syst Biol. 2007;3:121.

6. Messac A, Ismail-Yahaya A, Mattson CA. The normalized normal constraint method for generating the Pareto frontier. Structural and multidisciplinary optimization. 2003;25(2):86-98.
3.
